# Thyroid hormones reversibly inhibit metamorphic development in ophiuroid larvae

**DOI:** 10.1242/jeb.249351

**Published:** 2025-02-12

**Authors:** Elias Taylor, Jonathan D. Allen, Andreas Heyland

**Affiliations:** ^1^ University of Guelph, Integrative Biology, 50 Stone Rd East, Guelph, ON, Canada, N1G 2W1; ^2^ William & Mary, 540 Landrum Drive, Williamsburg, VA 23187, USA

**Keywords:** Thyroxine, Echinoderm, Metamorphosis, *Ophiopholis aculeata*, Larva, Endocrine system

## Abstract

The timing of metamorphosis and settlement is critical for the survival and reproductive success of marine animals with biphasic life cycles. Thyroid hormones (THs) regulate developmental timing in diverse groups of chordates, including the regulation of metamorphosis in amphibians, teleosts, lancelets, tunicates and lampreys. Recent evidence suggests a role for TH regulation of metamorphosis outside of the chordates, including echinoderms, annelids and molluscs. Among echinoderms, TH effects on development as well as underlying signaling mechanisms in early embryogenesis have been documented for echinoid (sea urchin) larvae, but we lack information on TH effects on metamorphic development in most other echinoderm groups, including the ophiuroids (brittle stars). Unexpectedly, we found that THs, principally 3,5,3′,5′-tetraiodo-l-thyronine (T4), reversibly inhibit metamorphic development and settlement in the daisy brittle star (*Ophiopholis aculeata*). Exposure to thiourea, an inhibitor of TH synthesis, accelerated metamorphic development. We showed that these effects were highly stage specific, providing evidence for a developmental point-of-no-return in ophiuroid metamorphic development. Furthermore, starvation of *O. aculeata* accelerated juvenile morphogenesis and settlement. Starvation also prevented the inhibitory effect of thiourea on TH function, suggesting that TH synthesis may play a role in delaying metamorphosis under conditions of high food availability. These findings provide evidence for a function of TH signaling in ophiuroid metamorphic development and suggest that exogenous TH sources may be involved in the regulation of metamorphic timing in *O. aculeata*. Together with new evidence of TH involvement in metamorphic development in a range of invertebrates, these findings further emphasize the versatile and central role of endocrine signaling in metamorphosis.

## Teaser

**Summary:** Thyroid hormones, principally thyroxine, reversibly inhibit metamorphic development in *Ophiopholis aculeata* larvae (an ophiuroid). This effect is stage-dependent and reversible.

## INTRODUCTION

Thyroid hormones (THs) control the timing of development and major life history events in many animals (Brent, 2012; Davis et al., 2016; Grimaldi et al., 2013; Pascual and Aranda, 2013; Taylor and Heyland, 2017). The most dramatic transition regulated by THs is metamorphosis, a post-embryonic process involving a range of physiological, developmental and ecological changes (Bishop et al., 2006a; Laudet, 2011). Throughout this paper, we will refer to metamorphic development, juvenile morphogenesis, metamorphic competence and settlement. In echinoderms, metamorphic development begins with juvenile morphogenesis in the larva, followed by metamorphosis and settlement. Juvenile morphogenesis is the development of juvenile features, which in echinoids, asteroids and ophiuroids occurs primarily in the juvenile rudiment. These features may include spines, tube feet, the hydrocoel and elements of the juvenile test. Metamorphic competence is the developmental stage at which a larva can settle (move to the benthos and attach to a substrate), likely determined by the presence of juvenile features developed during juvenile morphogenesis. A larva is ‘pre-competent’ or ‘post-competent’, depending on whether it has reached metamorphic competence.

Metamorphosis is widespread among animals, and most animals (including most vertebrates) metamorphose during their life history (Heyland and Moroz, 2006; Laudet, 2011). Some chordates undergo metamorphic development regulated by THs, including amphibians, teleost fishes, lancelets, tunicates and lamprey (Laudet, 2011). Notably in amphibians, TH signaling is both necessary and sufficient to regulate metamorphic development (Buchholz et al., 2011; Fu et al., 2018). Lamprey represent the singular example of THs repressing metamorphic development, with goitrogens (inhibitors of TH synthesis) stimulating early metamorphic development (Hoheisel and Sterba, 1963; Wright and Youson, 1977; reviewed in Manzon and Manzon, 2017).

TH regulation of metamorphic development is not limited to chordate deuterostomes and may be a shared feature of bilaterians, including molluscs, annelids and echinoderms (Carpizo-Ituarte, 1993; Chino et al., 1994; Eales, 1997; Fukazawa et al., 2001; Heyland et al., 2004; Holzer, 2015; Sainath et al., 2019; Saito et al., 1998; Taylor et al., 2023; Taylor and Heyland, 2017). The phylum Echinodermata comprises crinoids (sea lilies and feather stars), ophiuroids (brittle stars), asteroids (sea stars), echinoids (sea urchins and sand dollars) and holothuroids (sea cucumbers). In echinoids, THs have been shown to accelerate both juvenile morphogenesis and settlement (Armstrong and Lessios, 2015; Chino et al., 1994; Heyland et al., 2004; Saito et al., 1998; Taylor et al., 2024). Recent evidence from echinoids shows that THs act non-genomically, via an integrin membrane receptor (Taylor, 2017; Taylor et al., 2024), as well as genomically, via a nuclear hormone receptor (Saito et al., 2012; Taylor et al., 2024). In echinoids, TH binding sites in the gut and skeletogenic mesenchyme, and potential TH synthesis/sequestration in gut-proximal neurons, were identified (Taylor et al., 2024). Despite evidence for TH acceleration of metamorphic development in echinoids and asteroids (Chino et al., 1994; Heyland et al., 2004; Johnson and Cartwright, 1996; Saito et al., 1998), no studies have examined the function of THs in ophiuroid development.

**Fig. 1.**
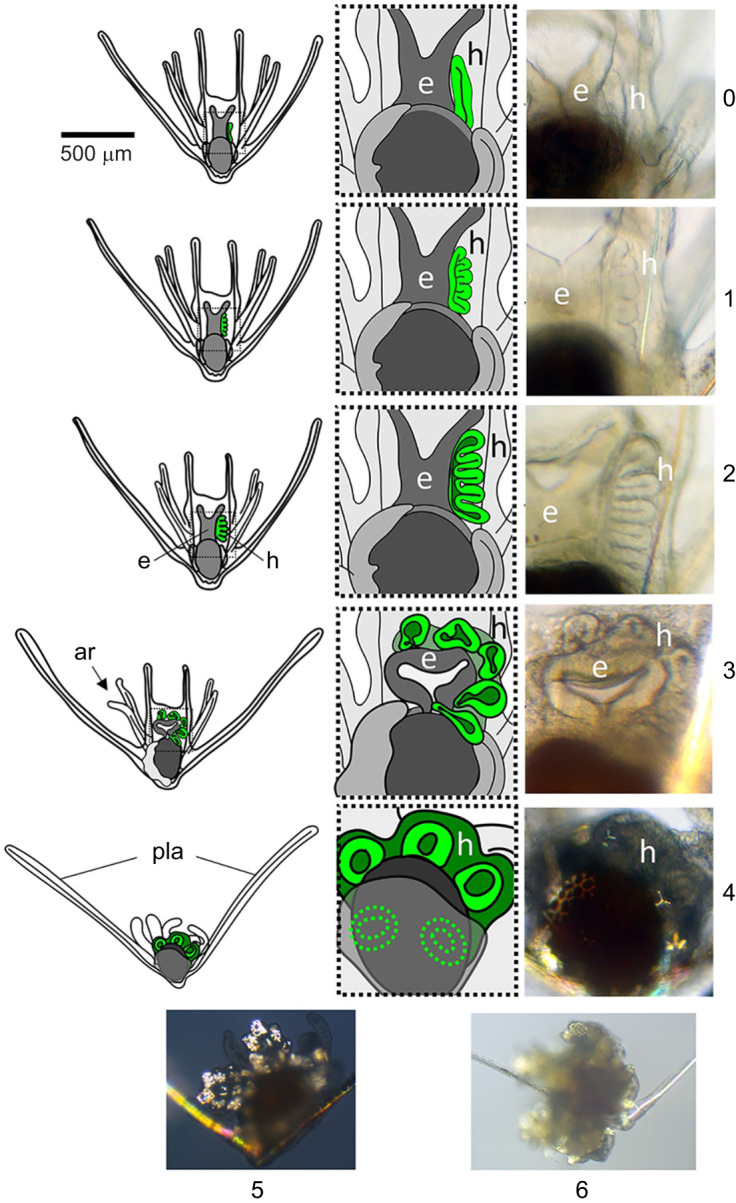
**Metamorphic development in *Ophiopholis aculeata* staged by hydrocoel morphology.** (0) Hydrocoel (green) has two distinct parallel layers. (1) Hydrocoel has begun forming five distinct lobes. Tissue thickness is roughly equal in the five lobes and base of the hydrocoel. (2) Hydrocoel lobes are extended. Base of the hydrocoel dramatically reduces in thickness. The oral lobe may be beginning to bend around the esophagus. (3) Hydrocoel is wrapping around the esophagus. Arm retraction has typically begun. (4) Hydrocoel has finished circularization, both ends of the hydrocoel have joined. The hydrocoel and rudiment are displaying clear pentaradial symmetry. (5) Rudiment has developed distinct and highly skeletonized arms. This is soon followed by separation of the juvenile from the posterolateral arms. (6) Larval arms are fully retracted. Juvenile is separated or separating from the posterolateral arms. h, hydrocoel; e, esophagus; ar, arm retraction; pla, posterolateral arms.

Indirect-developing ophiuroids (as well as echinoids and asteroids) prepare for settlement by developing a juvenile rudiment adjacent to the larval gut (Allen and Podolsky, 2007; Emlet, 2006; Mladenov, 1985; Narasimhamurti, 1933; Tominaga et al., 2004). *Ophiopholis aculeata* (daisy brittle star) juvenile morphogenesis begins with the formation of a five-lobed hydrocoel which wraps around the esophagus, acquires an additional tissue layer from the ectoderm, and forms the bulk of the newly metamorphosed juvenile (Fig. 1). Although most of the larval tissues are resorbed into the juvenile, the posterolateral arms in *O. aculeata* separate and may survive as a clonal larva (Balser, 1998). This mode of post-settlement larval arm separation is common in ophiuroids (Balser, 1998; Mladenov, 1979; Mortensen, 1921; Selvakumaraswamy and Byrne, 2006). Ophiuroids are not the only echinoderm larvae that undergo larval clonal reproduction (Allen et al., 2018; Bosch, 1988; Eaves and Palmer, 2003; Jaeckle, 1994; Knott et al., 2003); however, they are the only group in which larval cloning may occur post-settlement as a typical step in metamorphic development.

TH signaling may be intimately linked with feeding in echinoderm larvae. THs comprise a large portion of the echinoid larval diet (up to 2% by weight in algae; Chino et al., 1994; Heyland et al., 2006), and it has been previously proposed that exogenous TH might play a major role in regulating the timing of echinoid development (Eales, 1997; Heyland and Moroz, 2005; Miller and Heyland, 2010). Echinoderm metamorphic development is highly varied, with closely related species exhibiting divergent developmental modes (Hart et al., 1997, 2003; Jeffery et al., 2003; Kissinger and Raff, 1998; Wray, 1996). This variation on developmental mode is also linked to larval feeding modes in echinoderms, ranging from obligatorily feeding larvae to facultatively feeding larvae to non-feeding larvae (Allen and Pernet, 2007; McEdward, 1997; Reitzel et al., 2005). In facultatively feeding ophiuroid larvae, feeding in the plankton resulted in increased metamorphic success, larger juveniles and an increased juvenile survivorship (Nakata and Emlet, 2023). In perhaps the clearest example of the importance of TH signaling in determining the timing of life history transitions, TH exposure in an obligatorily feeding sand dollar larvae was sufficient to induce early metamorphic development and metamorphic competence, allowing for metamorphosis and settlement without feeding (Heyland et al., 2004; Heyland and Hodin, 2004). Although *O. aculeata* has an obligately feeding larva, increased food supply has been shown to impact the timing of metamorphic development, accelerating formation of the posterior coeloms and coelomic lobes (Strathmann et al., 2020).

Regulation of metamorphic timing is likely of crucial importance; a larva which metamorphoses precociously will have a lower survival rate as a juvenile (Hart and Strathmann, 1994; Havenhand, 1993; Nakata and Emlet, 2023; Pechenik et al., 1998; Werner, 1986). Metamorphosis allows organisms with complex life histories to exploit multiple ecological niches during development (Bishop et al., 2006a,b). In echinoderms, species with indirect-developing larvae undergo dramatic morphogenesis as a planktonic larva settles and metamorphoses into a benthic adult. The planktonic life of the larva allows for dispersal (Burgess et al., 2016) and, in species with feeding larvae, a chance to accumulate resources to improve the survival of the juvenile (Fadl et al., 2019; Richardson and Allen, 2023). However, planktonic life is rife with hazards, including unpredictable predation (reviewed in Vaughn and Allen, 2010) and food availability. Although these risks can be mitigated by investment in larval defenses (Vaughn, 2007) or larger larval feeding structures (Hart and Strathmann, 1994), every day spent in the plankton represents a high mortality chance, which is a trade-off that does not always pay out (Hart and Strathmann, 1994; Pechenik, 1990; Vaughn and Allen, 2010). The ophiuroid *O. aculeata* has a feeding larva with a pelagic period of 4 to 31 weeks (Strathmann, 1978). Timing of settlement and metamorphic development varies highly in *O. aculeata*, and in other echinoderms.

For larvae which settle to the benthos, settlement site selection increases the probability of finding suitable habitats. Most echinoderm larvae, including many echinoids and asteroids, are highly site-selective, relying on specific chemical and physical cues to settle and metamorphose on an appropriate surface in the benthos (reviewed by Doll et al., 2022). For some species, this means settling near conspecifics, at an appropriate depth, or near available juvenile food sources (Doll et al., 2022). Most ophiuroids, in contrast, are non-selective (Hodin et al., 2019). *Ophiopholis aculeata* will increase settlement rates in response to various cues, but will generally spontaneously settle and metamorphose (Hendler, 1991; Hodin et al., 2019; Selvakumaraswamy and Byrne, 2006). The mechanisms by which *O. aculeata* and other ophiuroids regulate metamorphic timing are unknown.

In this study, we sought to answer the question of whether THs regulate metamorphic development in ophiuroids. We addressed this question by examining inhibition of endogenous and exogenous TH sources and their impact on ophiuroid metamorphic development. Our results show that THs inhibit metamorphosis in *O. aculeata* larvae, and that this process appears to be dependent on both exogenous food supply and endogenous TH synthesis. Using our own staging scheme based on hydrocoel development in ophiuroid juvenile morphogenesis and settlement, we measured TH delay of metamorphic development. We also used echinoid larvae (*Strongylocentrotus purpuratus*) as a positive control for TH regulation of metamorphic development.

## MATERIALS AND METHODS

### Animal care

*Ophiopholis aculeata* (Linnaeus 1767) were collected by hand from the intertidal in Cobscook Bay, ME, USA, and shipped overnight to Williamsburg, VA, USA, to be spawned in late March 2021. Adults were induced to spawn either spontaneously via the disturbance of collection and shipping or through a simple procedure of repeated flipping of adult animals under bright lights and slowly warming water for 15–30 min, followed by a return to cold (12°C) water and dark conditions. Larvae at the early pluteus stage were shipped overnight on ice to the University of Guelph (ON, Canada) at 6 days post-fertilization (dpf). After transport, larvae displayed some desynchronization of development, which partially resolved over time. We used a developmental staging scheme to ensure synchronicity of all larvae in each experimental group. On arrival, ophiuroid larvae were transferred to 2 l glass beakers at a density of 0.5 larvae ml^−1^ (1000 larvae 2 l^−1^). Larval cultures were maintained at 14°C with salinity at 31–33 g l^−1^. Cultures were stirred constantly using a paddle system previously described by Strathmann (1987) and kept on a 12 h:12 h light cycle. The cultures were cleaned manually and had the water replaced with fresh artificial seawater (Instant Ocean) three times weekly. At the same time, cultures were fed *Rhodomonas salina* (UTEX LB 2763) at a density of 2000 cells ml^−1^ and *Dunaliella tertiolecta* (UTEX LB 999) at 3000 cells ml^−1^. Algae was cultured in F/2 medium before being centrifuged down and resuspended in filtered artificial seawater (FASW). Larval density was gradually reduced to 0.15 larvae ml^−1^ (300 larvae 2 l^−1^) over the course of 14 days by culture splitting and maintained at that level until the conclusion of the experiments.

Adult purple sea urchins (*Strongylocentrotus purpuratus*) were shipped from Monterey, CA, USA (2022–2023), where they were collected by diving, and subsequently kept in tanks of artificial seawater at the Hagen Aqualab (University of Guelph). The adults were fed a diet of kelp (*Macrocystis pyrifera* and *Kombu* spp.) every 2–3 days. Temperature was maintained at 14°C and salinity at 31 ppt. Sea urchins were spawned by injecting 0.5–1.5 ml of 0.5 mol l^−1^ KCl in distilled water, depending on the size of the sea urchin. Sperm was collected dry by pipetting sperm directly from the gonopores. Females were inverted over a beaker of FASW (0.2 µm filter) to collect eggs. After spawning, eggs were washed twice with FASW. Diluted sperm (approximately 1:100) was titrated into the beaker of eggs until fertilization success reached at least 90%. Fertilized eggs were washed once more with FASW to remove excess sperm and allowed to develop at 12°C in a 2 liter beaker until hatching. After 48 h at 12–14°C, hatched embryos were transferred to 2 liter beakers at a density of 1 larva ml^−1^. Sea urchin larval density was gradually reduced on a weekly basis to an approximate density of 0.25 larvae ml^−1^ (500 larvae 2 l^−1^). Cultures were fed thrice weekly, with *R. salina* at a density of 3000 cells ml^−1^ and *D. tertiolecta* at 4000 cells ml^−1^. Other details were similar to the *O. aculeata* culturing conditions.

### TH and inhibitor exposure conditions

THs (rT3, T4, T3; 3,3′,5'-triiodo-l-thyronine, l-thyroxine, 3,3′,5-triiodo-l-thyronine; Sigma-Aldrich T075, T1775, T2877) were prepared as described in Taylor and Heyland (2018) (dissolved in FASW with 1% DMSO; Sigma-Aldrich, D8418, at a working concentration of 10^−3^ mol l^−1^) and used at concentrations of 10^−7^ mol l^−1^, as in previous studies (Taylor et al., 2024). Thiourea (TU; Sigma-Aldrich, T7875), an inhibitor of TH synthesis (Anselmo, 2012; Heyland et al., 2006; Saito et al., 1998), and PD98059 (PD; Sigma-Aldrich P215), an inhibitor of MAPK (ERK1/2) phosphorylation, were also diluted to a working concentration of 10^−3^ mol l^−1^ in FASW and used at 10^−5^ and 5×10^−5^ mol l^−1^, respectively. DMSO was added to control, PD and TU groups to match the TH groups (1:100,000 DMSO:FASW). Six-well plates were prepared with 10 ml of FASW in each well. Hormone working stock was pipetted into each well to the indicated final concentration and exposed larvae were directly cultured in this hormone-containing medium. One larva was placed in each 10 ml well, for the purpose of tracking individual metamorphic development over multiple days.

### Developmental staging schemes

We developed a staging scheme for metamorphic development in *O. aculeata*, primarily relying on hydrocoel morphology (Fig. 1). The order of events in ophiuroid hydrocoel development appears to be common to many indirect-developing ophiuroids (MacBride, 1918; Mladenov, 1985; Narasimhamurti, 1933; Selvakumaraswamy and Byrne, 2006; Tominaga et al., 2004).

When scoring metamorphosis and settlement, *S. purpuratus* were considered to be metamorphosing if the rudiment was exposed with external tube feet and were considered settled if they had attached to the substrate. *O. aculeata* were considered to have metamorphosed and settled if they were attached to the substrate, with no visible larval arms and a juvenile morphology.

### TH and inhibitor exposures in *O. aculeata* larvae fed *ad libitum*

We tested the effect of THs on *O. aculeata* metamorphic development under typical culturing conditions – fed *ad libitum*. *Ophiopholis aculeata* were staged to stage 1, micrographed on an uncovered slide to prevent damage and randomly assorted to individual wells in 6-well plates in a 10 ml volume of FASW and either hormone or vehicle control. Larvae were fed *R. salina* daily (UTEX LB 2763) at a density of 2000 cells ml^−1^ and *D. tertiolecta* (UTEX LB 999) at 3000 cells ml^−1^. Larvae (*n*=12) were exposed to a vehicle control (1:100,000 DMSO:FASW), 10^−7^ mol l^−1^ T4, 10^−5^ mol l^−1^ TU, 5×10^−5^ mol l^−1^ PD98059, both T4 and TU, or both T4 and PD98059. TU is an inhibitor of TH synthesis and was used as a negative control to determine the effect of endogenous synthesized THs. PD98059, an inhibitor of MAPK phosphorylation, was used to test the MAPK-dependent non-genomic TH signaling pathway. The FASW and hormone exposures were replaced daily during feeding and micrography. We micrographed the larvae every 24 h over the course of 4 days for a total of five time points. Larvae were removed from their individual wells onto a slide for micrography in a minimum of FASW. After micrography, larvae were placed into a new plate with fresh FASW/hormone medium. Metamorphic development of each larva was scored according to our metamorphic staging scheme. A polarized light filter was used to track skeletogenesis and assist in staging the larvae.

### TH and inhibitor exposures in *O. aculeata* larvae under starvation conditions

Larvae were placed under starvation conditions to eliminate exogenous TH sources as a factor. After feeding, *O. aculeata* were starved for 3 days until the guts in all larvae appeared to be empty. After the 3 days under starvation conditions, the larvae were staged to stage 1 and exposed to a vehicle control (1:100,000 DMSO:FASW), 10^−7^ mol l^−1^ T4, 10^−7^ mol l^−1^ T3 10^−5^ mol l^−1^ TU, or both T4 and TU. During the observation period, larvae were also maintained under starvation conditions. Larvae were examined daily over 6 days for a total of seven time points. The experiment was otherwise identical to the *ad libitum* exposures.

### Removal of T4 and washing larvae after 5 days exposure

To test reversibility of TH inhibition of metamorphic development, we conducted an experiment where we divided larvae into two groups post-TH exposure and removed the THs from one group. After feeding, *O. aculeata* were starved for 3 days until the guts appeared to be empty in all larvae. The larvae (*n*=50) were staged to stage 1 and placed in 100 ml of FASW with 10^−7^ mol l^−1^ T4 for 5 days. From this group, larvae remaining at stage 1 were selected and washed three times in clean FASW to remove residual T4. Subsequently, larvae (*n*=12) were exposed to a hormone-free vehicle control (1:100,000 DMSO:FASW) or 10^−7^ mol l^−1^ T4, and development was recorded for 6 days.

### T4 exposure after hydrocoel wraparound has begun (stage 2)

To test whether the effects of THs were stage-specific, we exposed *O. aculeata* larvae after the most rapid metamorphic development had begun: post-stage 2. After feeding, *O. aculeata* were starved for 3 days until the guts appeared to be empty in all larvae. The larvae were staged to stage 2 prior to exposure. Larvae (*n*=12) were exposed to a vehicle control (1:100,000 DMSO:FASW) or 10^−7^ mol l^−1^ T4.

### *Strongylocentrotus purpuratus* metamorphic development

*Strongylocentrotus purpuratus* larvae (eight-armed stage) under the same exposure conditions (*n*=12, 10^−7^ mol l^−1^ T4) were used as a control (see Fig. 4). As has been previously demonstrated by Taylor and Heyland (2018), T4 exposure accelerates metamorphic development in *S. purpuratus*. After feeding, *S. purpuratus* were starved for 3 days until the guts appeared to be empty in all larvae. Larvae were staged to *S. purpuratus* soft tissue stage i–iii (Heyland and Hodin, 2014), micrographed with a compound microscope on an uncovered slide to prevent damage, and randomly assigned to individual wells in 6-well plates in a 10 ml volume of FASW. Larvae (*n*=12) were exposed to a vehicle control (1:100,000 DMSO:FASW), 10^−7^ mol l^−1^ T4, 10^−7^ mol l^−1^ rT3 or 10^−7^ mol l^−1^ T3. The FASW and hormone exposures were replaced daily. Larvae were examined daily over 7 days for a total of eight time points. They were staged under a microscope on an uncovered slide and metamorphic development (soft tissue and skeletal stages) of each larva was scored according to the staging scheme in Heyland and Hodin (2014). A polarized light filter was used to track skeletogenesis and assist in staging the larvae.

To test metamorphic competence, unfed larvae were placed into 6-well dishes in 10 ml of FASW at a density of 1 larva ml^−1^ and exposed to 10^−7^ mol l^−1^ T4, T3 or rT3, or were exposed to 10^−5^ mol l^−1^ thiourea for 144 h. Subsequently, larvae were exposed to 20 mmol l^−1^ KCl for 1 h to induce settlement, washed and placed back in clean FASW. After 24 h, larvae were examined for settlement and metamorphosis. Larvae were considered settled if they attached to the bottom of the dish with at least one external tube foot. Larvae were considered to be metamorphosing if they possessed an exposed rudiment and external tube feet.

### Statistical analysis

Effects of THs on developmental stage in ophiuroid and sea urchin larvae were tested with an ordinal regression in SPSS Statistics 29. Time in days post-exposure was used as a continuous independent variable, with metamorphic stages (Fig. 1) as the dependent ordinal variable. All experimental exposure groups (*n*=12) were compared with controls (*n*=12; unexposed larvae under the same condition). Statistical significance was determined by the Wald test. We report confidence intervals (CI), Wald statistics (*W*) and adjusted *P*-values. Metamorphosis and settlement rates in sea urchin larvae were evaluated with an ANOVA and *post hoc t*-tests. The rates of metamorphic development are reported as mean stages per day, with differences between groups reported as a change in the mean stages per day.

## RESULTS

### Developmental staging schemes

Prior to rudiment development (stage 0), the anterior left coelom, which will become the hydrocoel, had two distinct parallel layers and had not yet developed folds or lobes. At stage 1, the hydrocoel had begun forming five distinct lobes. These lobes formed the five main branches of the hydrocoel: the radial canals. The tissue thickness was roughly equal in the five lobes and base of the hydrocoel. At stage 2, the hydrocoel lobes were extended. The tissue at the base of the hydrocoel (adjacent to the esophagus) dramatically reduced in thickness in comparison with the five lobes. The anterior lobe began to bend around the esophagus in the first stage of hydrocoel wraparound. At stage 3, the hydrocoel wrapped around the esophagus. The five lobes began to form the first lateral buds, resulting in a pinched oval morphology. Arm retraction had typically begun in the postoral arms, and the posterodorsal/anterolateral arms may also have begun retracting. At stage 4, the hydrocoel had finished circularization with the anterior and posterior ends of the hydrocoel fusing into a continuous ring. At this point, the hydrocoel and rudiment were displaying clear pentaradial symmetry. After fusion of the anterior and posterior hydrocoel ends into a loop, the hydrocoel developed lateral buds, which formed the tube feet (sometimes called paired tentacles). Skeletal spicules were often forming in the rudimentary tube feet. By stage 5, the rudiment had developed distinct and highly skeletonized arms with multiple tube feet. This was typically soon followed by separation of the juvenile from the posterolateral arms and settlement. During stage 6, the larval arms were fully retracted. The juvenile ophiuroids settled and attached to the substrate and were separated or separating from the posterolateral arms.

*Strongylocentrotus purpuratus* rudiment development was staged using the staging scheme in Heyland and Hodin (2014). To briefly summarize, rudiment development was split into soft tissue development and skeletal development, which were scored separately. From soft tissue stage i–iii, the ectoderm invaginated to touch the hydrocoel. From soft tissue stage iv–viii, the hydrocoel developed five lobes, which elongated and curled to form a dome-like rudiment. This order was reversed relative to *O. aculeata* development, in which the hydrocoel forms lobes prior to ectodermal invagination. The skeletal stages 1–4 track tube foot skeletonization, followed by adult spine formation in stages 5–8, and the completion of tube foot skeletonization in stages 9–10.

### TH and inhibitor exposures in *O. aculeata* larvae fed *ad libitum*: THs delay metamorphic development

**Fig. 2.**
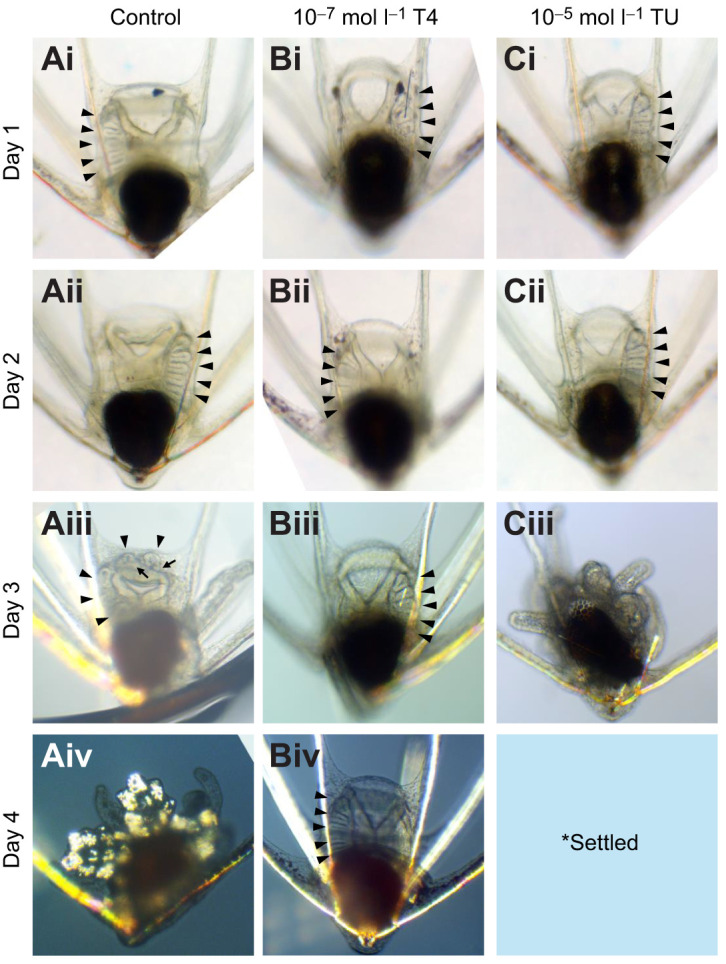
**Representative images of thyroid hormone-exposed *O. aculeata* pluteus larvae (fed *ad libitum*).** Polarized light microscopy is used on days 3 and 4 to reveal skeletal structures in the rudiment, and adjacent to the gut. Hydrocoel lobes are marked with arrowheads. (A) Development of control larvae was normal, with metamorphosis and settlement occurring over a 4–5 day period. In Aiii, the hydrocoel can be seen wrapping around the esophagus and developing tube foot buds (indicated with arrows). By Aiv, the ophiuroid has nearly completed metamorphosis and will shortly settle and discard the anterolateral arms (stage 5). (B) Thyroxine-exposed larvae (10^–7^ mol l^−1^ T4) displayed a reduced rate of metamorphic development, with hydrocoel development stalling at stage 1/2. Despite the reduced hydrocoel development, thyroxine-exposed larvae developed skeleton in the somatocoel adjacent to the gut (clearly visible in Biv). (C) Thiourea-exposed larvae (10^–5^ mol l^−1^ TU) metamorphosed and settled more rapidly than the control group. By day 3, the representative larva had a fused hydrocoel, and had nearly complete arm retraction (stage 4). No image is available for day 4, as the larva had settled and attached firmly to the plastic dish (stage 6). No abnormalities in the larvae or metamorphosed juveniles were observed.

**Fig. 3.**
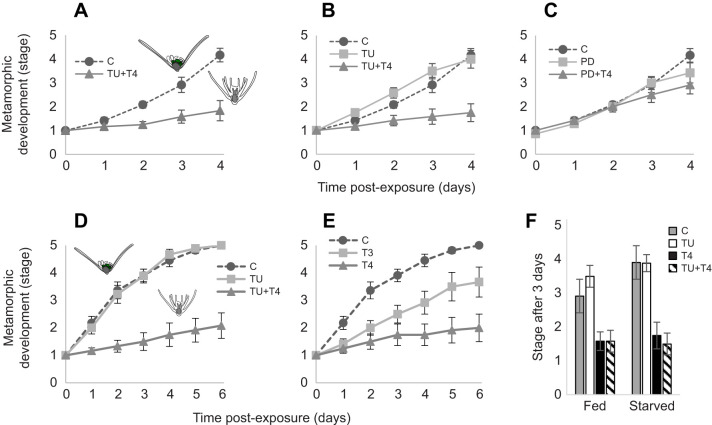
**Thyroid hormones inhibit metamorphosis in *O. aculeata* larvae under feeding and starvation conditions.** (A–C) In the first experiment, five groups of larvae (*N*=60, *n*=12) were fed *ad libitum* and exposed for 4 days to a vehicle control (C), thyroid hormone (T4), an inhibitor of thyroid hormone synthesis (TU) or an inhibitor of MAPK activity (PD98059), or to a combination of T4+TU or PD+T4. (D,E) In the second experiment, larvae were placed under starvation conditions to eliminate exogenous hormones as a factor. Larvae (*N*=60, *n*=12) were exposed for 6 days to a vehicle control, thyroid hormones (T4 or T3), an inhibitor of thyroid hormone synthesis (TU) or both TU and T4. (A) T4 inhibited metamorphic development in *O. aculeata* larvae. (B) TU slightly accelerated metamorphic development, whereas TU+T4 inhibits metamorphic development. (C) PD did not significantly inhibit metamorphic development, whereas PD+T4 inhibited metamorphic development. (D) Unlike fed larvae, starved TU-exposed larvae were not significantly different from the control larvae. T4 inhibited metamorphic development in *O. aculeata* larvae in both the presence and absence of TU. Starved larvae exposed to both TU and T4 were not significantly different from T4 alone. (E) In starved larvae, T4 and TU+T4 inhibited metamorphic development. In contrast, T3 inhibited metamorphic development. (F) During early observation (<3 days), starved larvae developed more rapidly to metamorphosis. This effect was reduced when comparing starved larvae with TU-exposed fed larvae. Error bars are ±s.e.m.

TH exposure inhibited metamorphic development, whereas thiourea-exposed larvae metamorphosed and settled more rapidly than the control group (Fig. 2). After a 4-day exposure to T4 (10^−7^ mol l^−1^), metamorphic development of *O. aculeata* was inhibited (Fig. 3A) by −0.60 stages day^−1^ (95% CI [−0.9, −0.3], *W*_1_=20.54, *P*=5.8E-06). This resulted in an average metamorphic stage of 1.83±0.46 (mean±s.e.m.), compared with the control at 4.17±0.28. By the end of the 4-day period, every control larva had either settled or was in the process of metamorphosing prior to settlement (12/12). In comparison, only 2/12 of the T4-exposed group settled, with the remainder not progressing further than stage 1 (8/12) or stage 2 (2/12).

The TU-exposed group (an inhibitor of TH synthesis; 10^−5^ mol l^−1^) also achieved settlement and metamorphosis at a proportion comparable to the control (Fig. 3B), with an average final stage of 4.00±0.37. Although similar numbers of TU-exposed larvae achieved metamorphosis, they did so more rapidly, by an average of 0.40 stages day^−1^ (95% CI [0.1,0.7], *W*_1_=6.01, *P*=0.014). Larvae exposed to both T4 and TU showed a similar inhibition of metamorphic development compared with T4 alone, inhibiting metamorphic development by −0.61 stages day^−1^ (95% CI [−0.9,−0.3], *W*_1_=21.02, *P*=4.5E-06; Fig. 3B).

PD98059 (an inhibitor of MAPK signaling) did not significantly inhibit metamorphic development (95% CI [−0.34,0.17], *W*_1_=0.46, *P*=0.50; Fig. 3C). However, it did inhibit settlement rates, with the average larva remaining at stage 3.43±0.38. PD98059 in combination with T4 inhibited metamorphic development by −0.22 stages day^−1^ (95% CI [−0.58,−0.03], *W*_1_=4.80, *P*=0.028).

### TH and inhibitor exposures in *O. aculeata* larvae under starvation conditions: TH synthesis plays a role in timing *O. aculeata* metamorphic development

Larvae were placed under starvation conditions to eliminate exogenous TH sources as a factor (Fig. 3). Unlike TU-exposed larvae fed *ad libitum*, TU-exposed larvae under starvation conditions were not significantly different from the control larvae under starvation conditions (95% CI [−0.1,0.1], *W*_1_=0.01, *P*=0.912; Fig. 3D). Starved larvae exposed to both T4 and TU were not significantly different from larvae exposed to T4 alone (95% CI [−0.1,0.1], *W*_1_=0.91 *P*=0.907). In starved larvae, T4 and TU+T4 inhibited metamorphic development in *O. aculeata* larvae by −0.60 stages day^−1^ (95% CI [−0.7,−0.5], *W*_1_=90.0, 88.8, *P*=2.4E-21, 4.3E-21).

In starved larvae, T4 and TU+T4 inhibited metamorphic development in *O. aculeata* larvae by −0.60 stages day^−1^ (95% CI [−0.7,−0.5], *W*_1_=90.0, 88.8, *P*=2.4E-21, 4.3E-21; Fig. 3E). In contrast, T3 inhibited metamorphic development by −0.26 stages day^−1^ (95% CI [−0.37,−0.15], *W*_1_=23.2, *P*=1.5E-06). During early observation (<3 days), starved larvae developed more rapidly to metamorphosis by 0.59 stages day^−1^ (95% CI [0.77,0.42]; Fig. 3F). This effect was reduced to 0.42 stages day^−1^ when comparing starved larvae with TU-exposed fed larvae (95%CI [0.57,0.26], *W*_1_=26.8, *P*=2.2E-07).

**Fig. 4.**
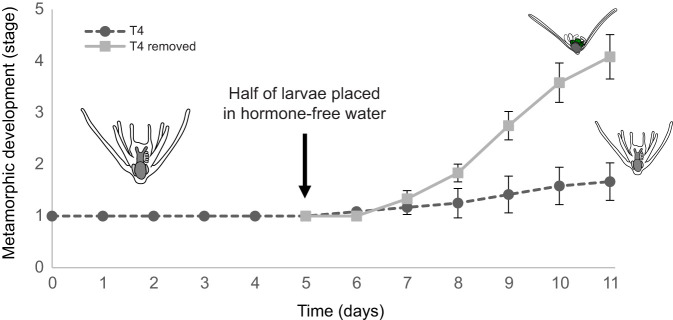
**Effects of T4 were reversible when larvae were placed in hormone-free water.** After 5 days of exposure to T4, larvae staged to stage 1 of metamorphic development were washed and moved to two separate conditions: with and without T4 (*N*=24, *n*=12). Larvae that were removed from the T4 underwent normal metamorphic development faster than the T4-exposed group. Error bars are ±s.e.m.

*Strongylocentrotus purpuratus* larvae under the same exposure conditions (*n*=12, 10^−7^ mol l^−1^ T4) were used as a control (Fig. 4). T4 accelerated both skeletal and soft tissue metamorphic development (95% CI [0.24,1.4], *W*_1_=7.7, *P*=0.006) in *S. purpuratus* rudiments.

Additionally, final rates of metamorphosis and settlement were measured in both *O. aculeata* and *S. purpuratus* larvae (Fig. S1). T4 significantly inhibited metamorphic development and spontaneous settlement rates in *O. aculeata* (*t*_22_=4.50, *P*=0.0002). In contrast, T4 increased both metamorphosis and settlement rates in *S. purpuratus* (*t*_58_=2.01, 2.64, *P*=0.049, 0.011) (Fig. S2).

### Removal of THs shows reversibility of delayed metamorphic development in *O. aculeata*

The effects of T4 were reversible when larvae were placed in hormone-free water (Fig. 4). Larvae that were removed from the T4 treatment underwent normal metamorphic development at 0.36 stages day^−1^ faster than the T4-exposed group (95% CI [0.22,0.51], *W*_1_=24.0, *P*=9.7E-07). Eighty-three percent (10/12) of previously exposed larvae resumed metamorphic development after 6 days, with 75% (9/12) completing settlement and metamorphosis. In contrast, 17% (2/12) remaining in T4-exposure conditions completed settlement and/or metamorphosis, with the remainder of larvae (10/12) unable to progress to hydrocoel wraparound (stage 2). All larvae that progressed to hydrocoel wraparound subsequently settled and metamorphosed.

### THs have no effect on metamorphic development post-hydrocoel wraparound in *O. aculeata*

**Fig. 5.**
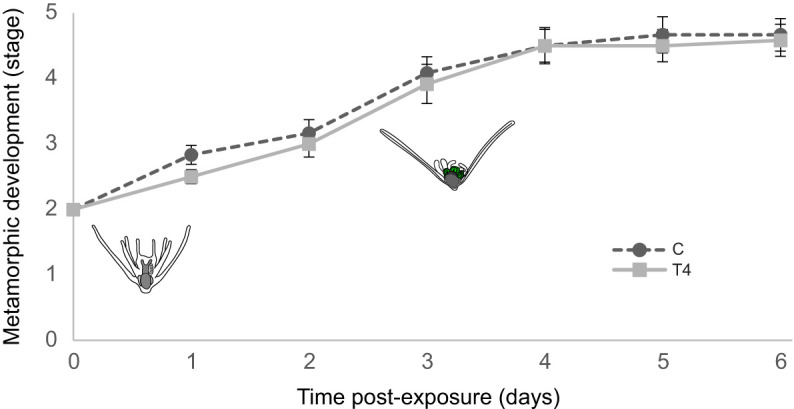
**Effect of T4 was not detected after metamorphic stage 2 (coelom wraparound).** Larvae were placed in either a vehicle control or T4 (10^−7^ mol l^−1^) at stage 2 and observed for 6 days (*N*=24, *n*=12). We did not detect a significant difference in development to metamorphosis in T4-exposed larvae that were exposed after stage 2 (coelom wraparound). Error bars are ±s.e.m.

We did not detect a significant difference in development to metamorphosis in T4-exposed larvae that were exposed after stage 2 (coelom wraparound; Fig. 5). T4-exposed larvae after stage 2 (*n*=12) developed an average of −0.07 stages day^−1^ slower relative to the control (95% CI [−0.18,0.04], *W*_1_=1.50, *P*=0.22). However, 92% of larvae in each group (11/12) successfully reached spontaneous settlement and metamorphosis (*t*_22_=0.00, *P*=1.00).

## DISCUSSION

### TH-induced delay of settlement in ophiuroids

We found that THs regulate the timing of metamorphic development in *O. aculeata* larvae. Specifically, THs delayed metamorphic development, a feature that has so far been described only in lamprey metamorphosis (Hoheisel and Sterba, 1963; Wright and Youson, 1977; reviewed in Manzon and Manzon, 2017). Thiourea, an inhibitor of TH synthesis, accelerated juvenile morphogenesis and settlement in ophiuroid larvae. However, this effect was not statistically significant when the larvae were placed under starvation conditions prior to exposure. These data suggest that TH synthesis in ophiuroid larvae may be important for delaying settlement under conditions of high food availability. Additionally, exogenous THs in the consumed algae may supplement endogenously synthesized hormones, as has been proposed in Eales (1997) and Heyland and Hodin (2004). The starved larvae underwent metamorphic development at an accelerated rate and settled more quickly than larvae fed *ad libitum*. We hypothesize that larvae under starvation conditions synthesize fewer THs (relative to fed larvae), resulting in accelerated metamorphic development. Accelerated metamorphosis under starvation conditions is particularly interesting in the context of a previous report that increased food supply accelerated metamorphic development (Strathmann et al., 2020). However, Strathmann et al. (2020) found that development was accelerated up to a lobed coelom, corresponding with our stage 1/2. Despite continued observation to 24 dpf, Strathmann et al. (2020) did not observe settlement, or development of a juvenile rudiment. There are two possible explanations for this differing result: either increased food supply accelerates development to stage 1/2 followed by a delay of metamorphosis, or a drastic change in food supply can induce early metamorphosis. To distinguish between these possibilities, it may be necessary to expose *O. aculeata* larvae to starvation conditions earlier in development, or to more gradually reduce the fed algae to zero.

*Ophiopholis aculeata* larvae exposed to T4 exhibited delayed metamorphic development regardless of being fed *ad libitum* or kept under starvation conditions. This similarity suggests that delayed metamorphic development and settlement in larvae fed *ad libitum* might be mainly attributed to TH regulatory control of metamorphic development. If the feeding effect were mainly attributable to another cause, such as increased nutrient reserves, we would expect to see starved larvae exposed to T4 metamorphose more rapidly than fed larvae exposed to T4. Alternatively, the metamorphic delay imposed by TH exposure may be strong enough to subsume any other effects of feeding or starvation.

The metamorphic delay was readily reversible after removing T4-exposed *O. aculeata* larvae from exposure conditions. However, larvae previously exposed to T4 underwent metamorphic development at a slower rate than larvae from the control groups. TH regulation of gene expression is a process that can take hours to days (e.g. Tata, 2006), and genetic inertia may account for the subsequent delay in metamorphosis. Alternatively, after an extended period of starvation and TH exposure, the larvae may lack the resources to develop the juvenile rudiment.

### Factors influencing ophiuroid development: larval cloning and food abundance

At least some ophiuroid larvae, including *O. aculeata*, employ an unusual reproductive strategy: larval cloning associated with metamorphosis and settlement (Balser, 1998). Larval cloning is widespread in echinoderms, with echinoids, holothuroids and asteroids displaying larval budding (Eaves and Palmer, 2003). Increased clonal reproduction associated with increased food availability has been previously demonstrated in echinoid and asteroid larvae (Vickery and McClintock, 2000; McDonald and Vaughn, 2010). During metamorphic development, ophiuroid larvae absorb the larval arms, with the exception of the posterolateral arms (Balser, 1998; Mladenov, 1979; Mortensen, 1921; Selvakumaraswamy and Byrne, 2006). The posterolateral arms are released into the water column after settlement, whereupon the arms develop into a feeding larva and are able to repeat the process, apparently indefinitely (Balser, 1998).

As clonal reproduction is uniquely associated with metamorphosis in ophiuroids, there is potential incentive for an ophiuroid larva to metamorphose as frequently as possible. Increased levels of TH – potentially signaling increased food availability in the plankton – lead to a greater time spent feeding prior to metamorphosis. An ophiuroid larva that devotes significant resources towards larval cloning shifts the optimal time of metamorphosis forward, as the expected potential benefit of feeding in the plankton might theoretically allow the survival rate to grow higher than 100%, if the single larva can produce multiple juveniles. This difference may partially account for the contrasting effects of THs on metamorphic development in ophiuroids and asteroids/echinoids.

Planktonic food availability is a major factor predicting the relative success of feeding versus non-feeding larval strategies (Marshall et al., 2018). Adjusting the planktonic feeding period in relation to food abundance, then, would help buffer larvae against uncertainty in food supply. For this reason, regulation of metamorphic timing is of crucial importance to the success of a marine planktotrophic larva. Ophiuroid larvae feeding on microalgae, which have been shown to contain THs or their precursors (Hernández Javier et al., 2018; Heyland and Moroz, 2005), may potentially ingest exogenous THs from consumed algae, signaling that the larval feeding strategy is successful, and delaying metamorphosis to accumulate further resources. Lack of food availability may indicate the end of the seasonal food abundance that feeding larvae take advantage of, requiring imminent settlement and metamorphosis.

We found that starvation accelerated ophiuroid metamorphosis; however, the opposite has been previously reported in some echinoids and ophiuroids (Hart and Strathmann, 1994; McAlister and Miner, 2018; Nakata and Emlet, 2023; Singh et al., 2018). For instance, rudiment resorption is a starvation response in echinoid larvae (Singh et al., 2018). The authors propose that in echinoids, the juvenile rudiment may provide a developmental buffer in the case of starvation. Under starvation conditions, the sea urchin larvae resorb the rudiment, and weather the poor food availability. In contrast, we hypothesize that ophiuroids may forgo accumulation of resources for larval cloning to metamorphose more quickly under starvation conditions. Reminiscent of the ‘desperate larvae’ hypothesis (Bishop et al., 2006b; Elkin and Marshall, 2007; Marshall and Keough, 2003), the ophiuroid larva may be more likely to adopt a suboptimal settlement strategy as energy stores decrease.

### Evolution of TH regulatory mechanisms in metamorphic development

There exists evidence for both nongenomic integrin-mediated TH signaling and genomic nuclear receptor-mediated signaling during echinoderm development. In echinoderm skeletogenic gastrulae (*S. purpuratus* and *O. aculeata*), THs bind principally to the membrane of skeletogenic mesenchyme cells (Taylor and Heyland, 2018; Taylor et al., 2024). However, in echinoderm larvae which have begun juvenile morphogenesis, including *S. purpuratus*, *O. aculeata* and *Pisaster ochraceus*, THs bind to the nucleus, perinuclear region, and membrane of gut and rudiment tissues (Taylor et al., 2024).

Although TH-mediated gene regulation has not been investigated in ophiuroids, THs have been shown to regulate gene expression in echinoid larvae via the nuclear TH receptor (i.e. via a genomic mechanism; Taylor et al., 2023). During juvenile morphogenesis, echinoid larvae (*S. purpuratus*) show increased levels of gene regulation in areas proximal to TH response elements in the genome (Taylor et al., 2023), as well as T4 binding in the nuclear protein fraction (Saito et al., 2012). Taylor et al. (2024) and Wynen et al. (2022) found differential effects of THs pre- and post-onset of metamorphic development, suggesting echinoid larvae undergoing juvenile morphogenesis present additional or changed TH receptor responses relative to during larval development. After onset of juvenile morphogenesis, then, echinoderm larvae likely become capable of responding to TH signals via the nuclear TH receptor.

It is not known whether ophiuroid larvae synthesize THs; though they possess the required pathways and are responsive to inhibitors of TH synthesis. However, TH levels rise in echinoid larvae prior to metamorphosis (Chino et al., 1994), suggesting a physiological relevance to expression of nuclear TH receptor during echinoderm metamorphic development.

Additionally, inhibiting MAPK signaling appeared to partially rescue the effect of T4 exposure in *O. aculeata* larvae, suggesting that the mechanism of T4 actions are at least partially MAPK-dependent. It should be noted that inhibiting MAPK phosphorylation also resulted in delayed settlement and an increased mortality rate. The available evidence indicates that TH signaling via both a membrane receptor and nuclear hormone receptor may cooperate to regulate metamorphic development in *O. aculeata*, as in other distantly related echinoderms including *S. purpuratus* and *P. ochraceus*.

Given TH acceleration of metamorphic development in echinoids, vertebrates and molluscs, it is unexpected that *O. aculeata* responds in an inverse fashion. Lampreys represent another life history in which THs repress metamorphosis (Manzon and Manzon, 2017). Similarly to *O. aculeata*, inhibition of TH synthesis is sufficient to induce or accelerate metamorphic development in lamprey, and exposure to THs (principally T3, the primary TH in vertebrates) inhibits and delays metamorphic development. However, lampreys have various ecological and developmental differences with ophiuroids. Similar to ophiuroids, lamprey undergo extensive gut remodeling and lose access to the larval food supply (Manzon and Manzon, 2017). It has been hypothesized that high levels of THs function in lampreys to delay onset of metamorphosis until the larva has accumulated energy reserves sufficient for metamorphic development (Manzon and Manzon, 2017).

In this case, there may be a common factor between lampreys and ophiuroids that could have driven convergent evolution of TH inhibition of metamorphic development: the need to prepare energy reserves. Accumulation of energy reserves is critical for the survival and reproductive success of ophiuroids with feeding larvae, perhaps more so than other echinoderms. Larvae that possess sufficient reserves have a higher juvenile survival rate (Nakata and Emlet, 2023) and may additionally undergo clonal reproduction (Balser, 1998).

Multiple possible evolutionary trajectories could allow for TH promotion of a gene or gene regulatory network to be replaced with inhibition. Briefly, the TH response elements in the genome to which the nuclear receptor binds may have been duplicated, deleted or mutated such that the nuclear receptor recruits corepressors blocking gene expression. Alternatively, the nuclear TH receptor may have mutated such that it recognizes a different repeat spacing, affecting the gene expression of many genes simultaneously. This has occurred many times during nuclear receptor evolution (Huang et al., 2015; Taylor et al., 2023; Taylor and Heyland, 2022), and crosstalk between nuclear receptors at a common response element can result in a reversed function of the response element (Glass et al., 1988). Nuclear hormone response elements are highly similar and lead to complex interactions between nuclear hormone receptors, including thyroid hormone receptor (THR) (Helsen et al., 2012; Liu and Brent, 2010). Another possibility is that a downstream transcription factor regulated by THs may have acquired a function delaying metamorphic development. A final possibility is that THs may inhibit metamorphic development by a non-genomic function, either via extra-nuclear actions of THR (Davis et al., 2016; Taylor and Heyland, 2022) or via another receptor entirely, such as a membrane integrin (Bergh et al., 2005; Davis et al., 2016; Taylor and Heyland, 2018; Taylor et al., 2024).

### Conclusions

THs, principally thyroxine, reversibly inhibit metamorphic development in the ophiuroid *O. aculeata*. This effect is highly stage-dependent, and TH exposure prior to hydrocoel wraparound and circularization is ineffective. Additionally, the effect is reversible: larvae removed from TH exposure conditions developed through metamorphosis and settlement with no abnormalities. The mechanism of action appears to be at least partially MAPK-dependent, as in echinoids; however, previous work also shows nuclear binding sites. TH regulation of metamorphosis is the opposite of what has been found in other bilaterians, with the sole exception of lamprey. The metamorphic delay induced by THs may be a result of the unusual aspects of ophiuroid metamorphic development and settlement, including: larval cloning associated with metamorphosis, a lowered investment in pre-settlement juvenile morphogenesis, and a non-selective settlement strategy. Our data suggest that TH signaling in ophiuroid larvae delays settlement under conditions of high food availability. Mechanisms underlying this change in TH action are still unclear and bear further investigation.

## Supplementary Material

10.1242/jexbio.249351_sup1 Supplementary information
