# Supplementary material for: Thyroid hormones reversibly inhibit metamorphic development in ophiuroid larvae
Source: J Exp Biol. 2025 Feb 12;228(3):JEB249351. doi: 10.1242/jeb.249351 (PMC11883245; doi:10.1242/jeb.249351)
Supplement: Supplementary information [file jexbio-228-249351-s1.pdf]

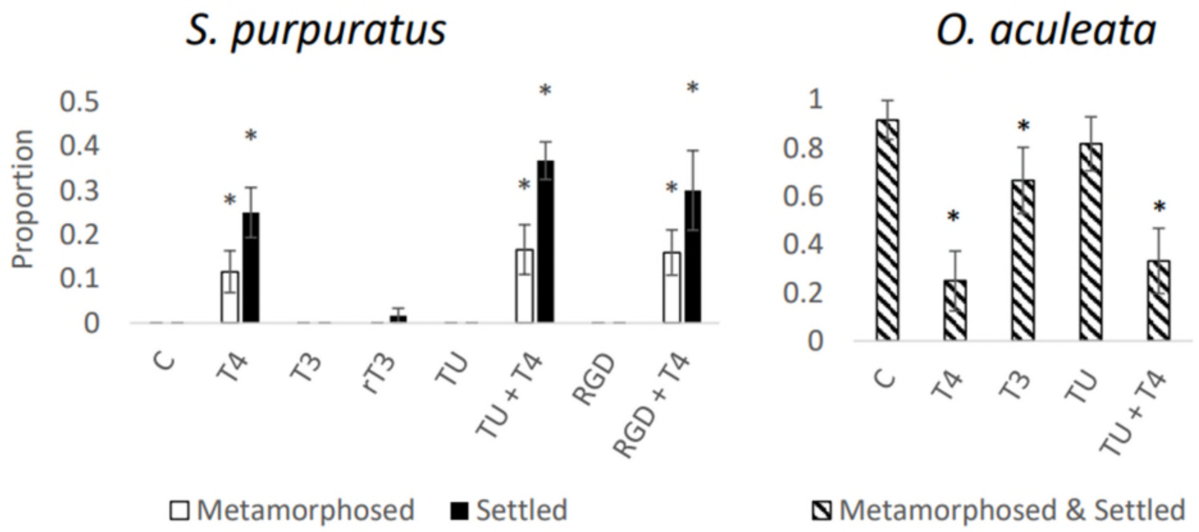

Fig. S1. T4-exposed *S. purpuratus* metamorphose and settle at higher rates on exposure to a settlement cue: KCl. *O. aculeata* metamorphosis spontaneously in the absence of a cue, but T4-exposure inhibits metamorphic development to settlement. *S. purpuratus* were considered to be metamorphosing if the rudiment was exposed with external tube feet and were considered settled if they had attached to the substrate. *O. aculeata* were considered to have metamorphosed and settled if they were attached to the substrate with no visible larval arms. Statistical significance relative to the control ($p < 0.05$) is indicated with *. Error bars are ± SE.

Journal of Experimental Biology • Supplementary information

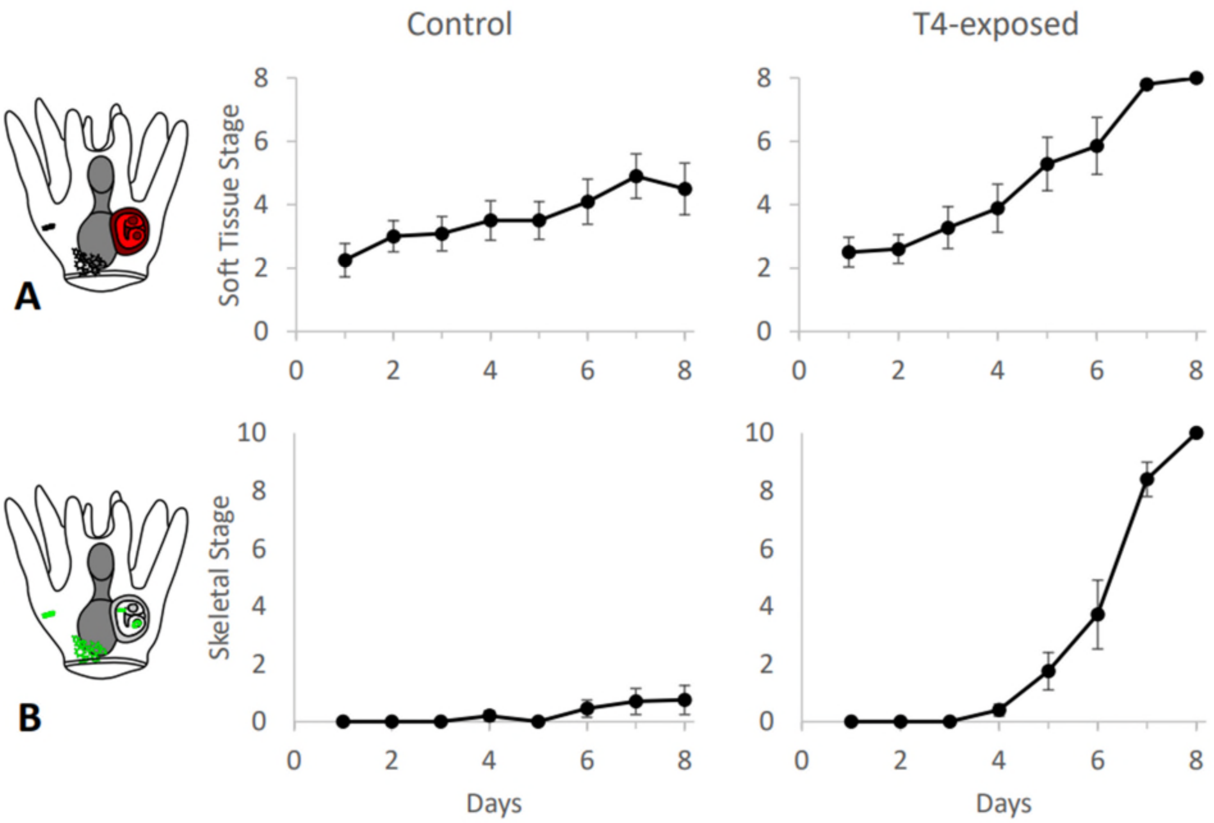

Fig. S2. T4 exposure under the same conditions accelerates development to metamorphosis in *S. purpuratus.* Here we exposed *S. purpuratus* plutei at the eight-armed stage to the same conditions as *O. aculeata* (Fig 1., n = 12, 10^{-7} M T4). T4 accelerated both (A) skeletal and (B) soft tissue (95% CI [0.24,1.4], W(1) = 7.7, p = 0.006) in *S. purpuratus* rudiments. Error bars are ± SE.

Journal of Experimental Biology • Supplementary information
